# Survival Analysis and Failure Modes of Total Hip Arthroplasty Using a Cemented Semi-Retentive Acetabular Cup

**DOI:** 10.3390/jcm12247506

**Published:** 2023-12-05

**Authors:** Gabriel Stan, Mihai Dan Roman, Horia Orban, Vlad Alexandru Georgeanu, Rares Stefan Deculescu, Lacramioara Aurelia Brinduse, Nicolae Gheorghiu

**Affiliations:** 1 Faculty of Medicine, “Carol Davila” University of Medicine and Pharmacy, 050474 Bucharest, Romania; gabriel.stan@umfcd.ro (G.S.); vgeorgeanu@hotmail.com (V.A.G.); nicolae.gheorghiu@umfcd.ro (N.G.); 2 Faculty of Medicine, University “Lucian Blaga” of Sibiu, 550024 Sibiu, Romania

**Keywords:** constrained, dislocation, failure, survival, total hip arthroplasty

## Abstract

To reduce the incidence of total hip revisions, there have been continuous efforts to enhance prosthetic materials and designs to optimize implant survival. A primary implant with a constrained acetabular component is often used to minimize the risk of dislocations even though this approach has some drawbacks as reported in the literature. To address these concerns, this study aimed to assess the survivorship and dislocation rate of a semi-retentive cemented acetabular cup when used as a primary implant. The specific cemented cup that we studied was not present in any study that we consulted, so to fill this gap, we conducted a retrospective examination of 527 cemented hip prostheses that utilized the semi-retentive cup between the years 2005 and 2012. We employed Cox multiple regression models for our statistical analysis. The revision due to dislocation occurred in 12.8% of all cases, with a lower incidence of 5% (14 cases) in age groups >70 years than in age groups <70 years (14%—32 cases) (*p* < 0.001). The survival rates of the semi-retentive cemented acetabular cup were 98.6% (520 cases) at 5 years and 92.2% (487 cases) at 10 years. The survival rates were significantly lower in women than men, with 1.9% (7 cases) toward 0% at 5 years and 8.1% (30 cases) toward 5% (7 cases) at 10 years (*p* = 0.002). The difference in failure rates between age groups over 70 years (2.3%—10 cases) and age groups under 70 years (11.5%—34 cases) was also statistically significant (*p* < 0.001). Our study indicates that the semi-constrained design may cause frequent damage to the polyethylene liner due to impingement and wear, which are the primary factors for failure. Also, this implant has a similar risk of revision due to dislocation as reported in studies and may be beneficial as a primary implant in elderly patients with low-demanding lifestyles, muscular insufficiency, and low compliance regarding hip prosthetic behavior, without a major effect on survivorship.

## 1. Introduction

Dislocation is a relatively frequent complication following total hip arthroplasty, with most incidents occurring in the early postoperative period. The reasons for dislocation may be attributed to either patient-related factors, such as previous surgeries, spine fusion surgery, or neurological impairment, or to surgeon-related factors, including approach, the orientation of implants, and impingement. About one-third of cases require surgical treatment, which is much more intricate and technically challenging than the primary surgery, with longer operating times and increased blood loss, potentially leading to a longer hospital stay. Effective management of postoperative dislocation after total hip arthroplasty requires prompt recognition and intervention. In addition to surgical techniques, patient education and compliance with postoperative restrictions play a significant role in reducing the risk of dislocation. As the population ages and the demand for total hip arthroplasty continues to increase, ongoing efforts to improve surgical techniques and implant design will be critical in minimizing the risk of dislocation and improving patient outcomes. Various measures can be implemented to prevent dislocation, such as the use of constrained liners, elevated rim liners, dual mobility implants, or trochanteric advancement. To reduce the incidence of total hip revisions, ongoing efforts have been directed toward the refinement of prosthetic materials and designs with the overarching goal of optimizing implant survival. Notably, the deployment of a primary implant featuring a constrained acetabular component stands out as a prevalent strategy employed to mitigate the risk of dislocations, a complication with significant implications for patient outcomes and healthcare resource utilization.

Despite the widespread adoption of this approach, it is imperative to acknowledge that, as documented in the existing literature, there are reported drawbacks associated with the utilization of primary implants featuring constrained acetabular components. These drawbacks necessitate a thorough exploration to delineate their specific nature and potential impact on clinical outcomes. By systematically scrutinizing these reported limitations, a comprehensive understanding can be achieved, contributing valuable insights to the ongoing discourse surrounding the optimization of total hip arthroplasty interventions.

In light of the intricate interplay between implant design, material properties, and patient-specific factors, a nuanced evaluation becomes essential for elucidating the complex dynamics governing the success of primary hip implants. As orthopedic practitioners strive to strike a balance between minimizing dislocation risks and addressing associated drawbacks, a meticulous examination of both the advantages and limitations of specific implant configurations becomes integral to informed decision making and the continual evolution of orthopedic practice. A primary implant with a constrained acetabular component is often used to minimize the risk of dislocations. However, this approach has some drawbacks as reported in the literature.

Various techniques have been proposed to prevent primary dislocations in prosthetic hips. Proper implant positioning is crucial to ensure the stability and longevity of the prosthesis post-surgery. Despite correct surgical techniques, dislocations can still occur due to several factors such as neuromuscular conditions, patient activity levels, prosthetic design, and patient demographics including advanced age, female gender, and high BMI. Other factors like the specific hip anatomy, trochanteric nonunion, and abductor insufficiency can also contribute to dislocation occurrences. Also, bearing surfaces can play a role in hip revisions due to dislocation. According to Castagnini [1], the mean, ceramic-on-ceramic design had a lower risk of revisions due to dislocation than ceramic-on-polyethylene.

The dislocation rate in primary arthroplasty ranges from 0.3% to 10% [2,3], but this risk can be minimized by careful patient selection and implant choice. One approach to reduce the risk of dislocation is to use a constrained acetabular component as a primary implant in selected cases. However, there are also disadvantages associated with this method. The constrained design mechanically limits the range of motion and increases the risk of impingement [4], and there are concerns about polyethylene wear and increased forces on the bone–prosthesis interface [5]. There are some benefits described in using a tripolar-constrained liner. It was demonstrated that they provide improved survivorship compared to locking ring types [6].

The Coriolis semi-retentive cemented acetabular cup, a product of Fournitures Hospitalieres (Heimsbrunn, France), was designed to achieve stable fixation in total hip arthroplasty. Its semi-retentive design should minimize the risk of prosthetic dislocation during ambulation and other daily activities. Customization of the shape and size of the semi-retentive cemented acetabular cup can be performed to achieve anatomical compatibility and optimize clinical outcomes for individual patients undergoing total hip arthroplasty.

The surgical procedure may be technically demanding, and successful outcomes rely on the expertise of a skilled hip replacement surgeon with adequate experience in this technique. The semi-retentive cemented acetabular cup has been associated with favorable clinical outcomes, including the reduced risk of dislocation, improved implant survival, enhanced patient mobility, improved clinical outcomes, and reduced revision rates.

There are studies reporting the survivorship and failure modes of total hip arthroplasty using cemented semi-retentive acetabular cups, but none have specifically evaluated the Coriolis semi-retentive cemented acetabular cup when used as a primary implant. Our study aims to fill this research gap by analyzing the survivorship and dislocation rate as a cause of failure for this type of semi-retentive cemented acetabular cup when used as a primary implant. The novelty consists in the evaluation of this particular acetabular cup and its failure modes and, additionally, may help in the decision-making process when a constrained implant is needed, regarding the patient’s age and gender. This study was based solely on revision rate.

## 2. Materials and Methods

**Figure 1:**
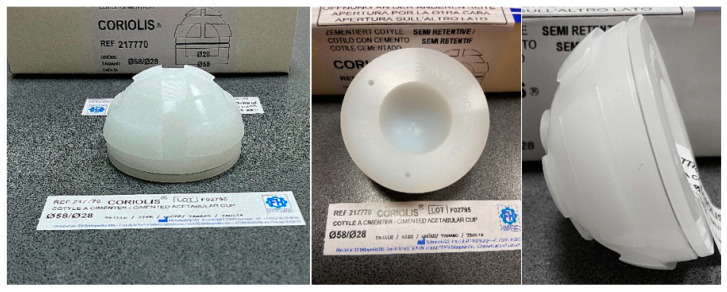
Coriolis cemented cup.

**Figure 2:**
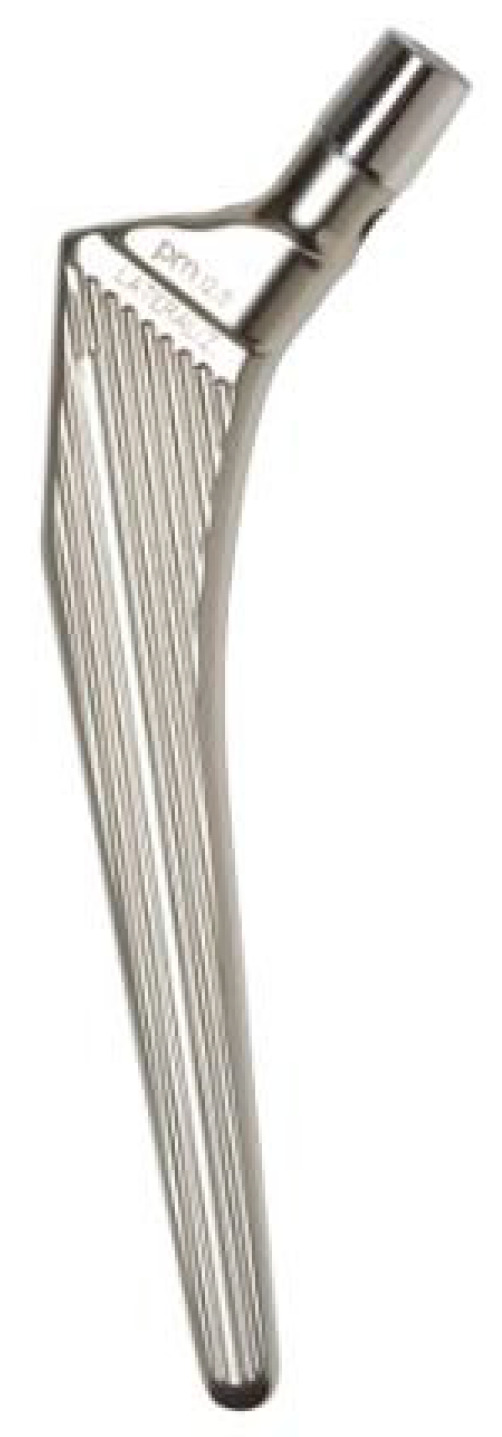
Permedica femoral component.

A total of 527 cemented hip prostheses using a semi-retentive cup, implanted at Elias University Hospital, Bucharest, between 2005 and 2012, was retrospectively examined. The demographic details of the patients like patient gender, age, and affected side were recorded. This study received approval from the Ethics Committee with certificate nr 5265 (Dr Negoita Silvius, Anca Cirjeu). Most cases (85.5%) had a primary diagnosis of osteoarthritis, while the remaining cases were attributed to osteoarthritis secondary to hip dysplasia (4%), avascular necrosis of the femoral head (2.7%), post-traumatic osteoarthritis (5.7%), and rheumatoid osteoarthritis (2.1%). The Coriolis (Figure 1) cemented semi-retentive UHMWPE polyethylene acetabular cup (Fournitures Hospitalieres, France) was used in all patients primarily due to its economical nature and availability. It is sterilized using ETO and comes in internal diameters of 22.2–28 and 32 mm, and includes a snap-action mechanism when introducing the head into the cup. The femoral component used in all cases was the cemented “Self-Locking Femoral Stem”, and the 28 mm femoral head was made of CoCr alloy (Permedica, Merate L.C., Italy). The self-locking stems for cemented application are made of highly nitrogenized stainless steel forged alloy. The anterior and posterior surfaces present longitudinal grooves that favor cement anchoring both in the metaphyseal and diaphyseal area (Figure 2). A cement without antibiotics (Groupe Lepine, Genay, France) was utilized. The surgeries were conducted by a single surgeon using a lateral approach, and the implantation of the components adhered to the accepted guidelines for hip implant orientation, with an optimal position defined as a 40° inclination ±10° and 15° of anteversion ±10°. The acetabulum was reamed till that bleeding cancellous bone was exposed. The bone surface was cleaned with saline lavage and then dried. The cement was then placed into the acetabulum and pressurized over the bone surface. The acetabular component was then inserted in the ideal orientation using an introducer. After the procedure, the patients began physical therapy, full weight bearing with restricted adduction, inward rotation, and flexion for the first three months, starting the day after surgery. The acetabular bone loss was evaluated and classified using the Paprosky classification on plain radiographs. We conducted survival analysis, taking into account patient death as a competing event. Our analysis considered endpoints such as revision for any reason or aseptic reasons as endpoints. The revision rate and mortality rate were assessed based on institutional records and the National Register of Hip Implantation. Kaplan Meier curves were used for survival analysis, and a *p*-value of <0.0001 was considered statistically significant. Age, sex, and diagnosis were adjusted in the Cox multiple regression models. The endpoint for survival was the revision of the entire implant or its components.

## 3. Results

The study’s demographic characteristics are presented in Table 1. 

**Figure 3:**
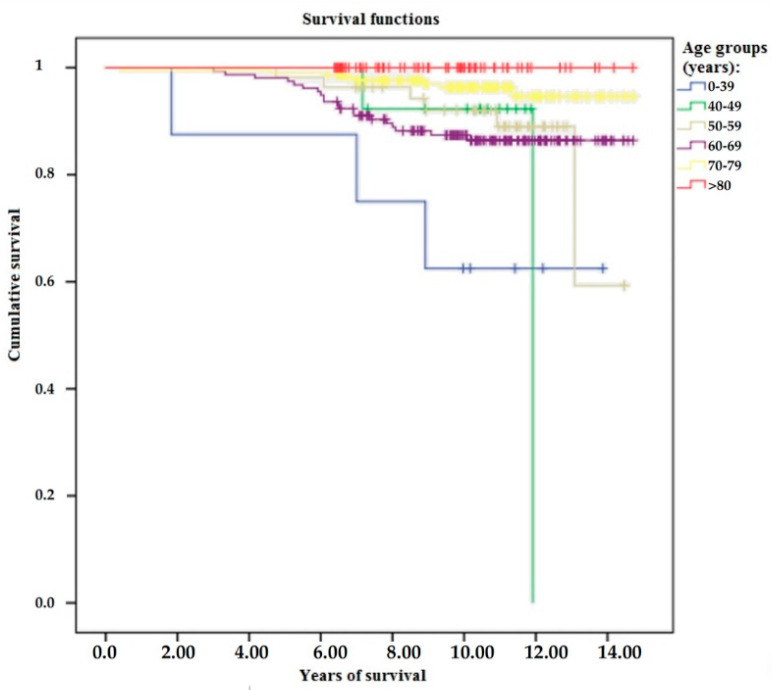
Survival results according to age groups.

The dislocation rate as a reason for revision was found to be 12.8% of all cases, which is different from the cohort dislocation rate. The dislocation rate as a reason for revision in age groups above 70 years was approximately 5%, which is significantly lower than in age groups below 70 years (14%) (*p* < 0.001). The implant’s failure rates were 1.4% (7 cases) at 5 years and 7.8% (41 cases) at 10 years. Upon analyzing by gender, the rates were worse in women than men, with 1.9% (7 cases) toward 0% at 5 years and 8.1% (30 cases) toward 5% (7 cases) at 10 years. Analyzing by age groups, there was a significant difference between groups above 70 years, with 2.3% (10 cases) failure rates, and groups below 70 years, with 11.5% (34 cases) failure rates (*p* < 0.001). The results are depicted in Figure 3. 

**Figure 4:**
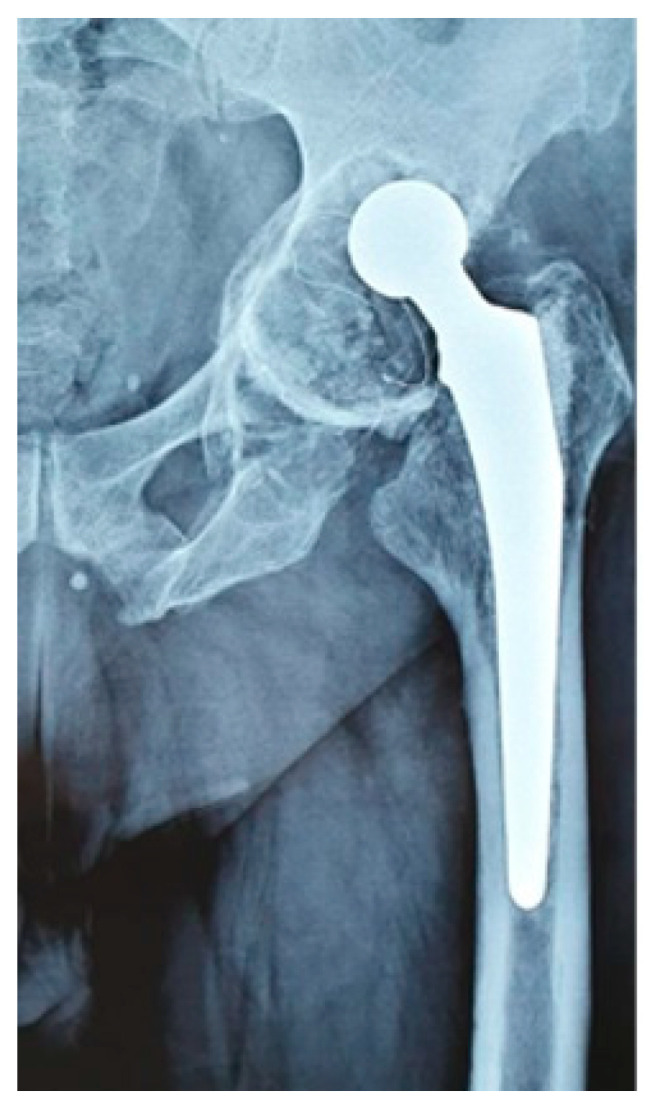
Massive acetabular bone loss with cup loosening in a failed Coriolis case.

A total of 39 revisions were identified due to various causes, with 10% of the revisions being classified as Paprosky I, 35% as Paprosky II (II A-35%; II B-20%; II C-45%), and 55% as Paprosky III (III A-85%; III B-15%) (Figure 4). 

The main cause of failure was acetabular cup loosening with backside wear (65%), followed by femoral loosening (15%) and dislocations (12.8%). The causes for revision are summarized in Table 2. Patients who deceased during the follow-up period after 31 December 2012 were treated as censored at that juncture. Their data were integrated into the analysis up to that specific time point. Out of the initial cohort, 21 patients were lost to follow-up, and their data were included up to their last clinical and radiographic evaluation. The mean follow-up duration was 10 ± 1.5 years (range: 5–15). 

## 4. Discussion

Our study found that the constrained acetabular cup had a similar risk of revision due to dislocation as reported in primary total hip arthroplasty studies. Additionally, in the elderly population, the re-dislocation rates were even lower. On the other hand, our findings suggest that the semi-constrained design often results in damage to the polyethylene liner due to impingement and wear, which are the primary factors leading to failure. These damage mechanisms typically occur in conjunction with migration, abrasion, and, in some cases, fracture of the constraining ring. 

According to Noble [7], the failure of the locking ring was responsible for 51% of failures in cases where constrained cups were used for hip revisions, whereas 28% of revisions were due to acetabular cup loosening, 6% due to backside wear, and 22% due to infection. The study concluded that the failure of the locking liner ring and loosening of the acetabular cup were the primary causes of mechanical failure with constrained liners, and that polyethylene is an inadequate material for restricting hip motion to prevent instability. The forces that cause dislocation in unconstrained cups are transferred to the rim and the shell of the constrained component in cases of major impingement, leading to damage to the polyethylene cup and possible overloading of the prosthesis–bone interface. 

Yun et al. [8] conducted a retrospective study on 29 patients who experienced failure of the constrained acetabular construct. The analysis revealed four distinct modes of failure, including fixation failure to the pelvis, liner dissociation, biomaterial failure, and femoral head dislocation. The findings suggest that constrained liners are particularly vulnerable to mechanical overload and that the risk of failure can be mitigated by reducing prosthetic impingement and avoiding technical errors. Biomaterial failure refers to unintended wear or fracture of the liner or retaining ring. 

Studies that have evaluated constrained implants used in revision settings have shown re-dislocation rates ranging from 29% to 50% [7,9]. Constrained acetabular components are commonly used in hip replacement surgeries where the patient has a history of instability or dislocation. The findings reported by Yun et al. highlight the importance of carefully considering the potential failure modes of these components and monitoring patients who have received them for signs of complications. 

A study conducted in Finland evaluated 373 cases of primary total hip arthroplasty (THA) where constrained acetabular implants were used between 2006 and 2017. The risk of revision due to dislocation in the group that received constrained acetabular devices as primary implants was found to be similar to the risk observed in the reference group [10]. The 8-year survivorship (97%) of the constrained acetabular liner group was equal to that of the reference group revision for any reason as the endpoint and was a bit higher than in our study (94.7% at 10 years). The study results were stratified by age groups to explore the reasons for failure, which may be particularly relevant for understanding outcomes in elderly patients or those with impaired neurological status, as most previous studies have reported on the use of retentive cups in these populations. The results of the Finnish study suggest that constrained acetabular devices may be a viable option for reducing the risk of dislocation in primary THA. 

Hip implant survivorship is influenced by various factors, such as patient characteristics, implant design, and surgical technique. Several studies have reported 10-year survival rates with revision for any reason of 98.4%, 95.6%, and 87.9% for metal-on-poly, ceramic-on-ceramic, and metal-on-metal designs, respectively [11]. Another study reported a 20-year survivorship of approximately 97% for cemented hip implants [12]. In the Finnish study, the 8-year survivorship for any reason was 94% (confidence interval [CI]: 91–96) for the constrained implant group and 93% (CI: 89–97) for the reference group [10]. The use of another type of cemented retentive cup, the Lefèvre cup, has been associated with a 10-year survival of 89% (CI, 83–94). A 10-year survival rate of 92.4% was reported for cup revision due to aseptic reasons in primary and revision cases using this implant [13]. The overall 10-year survivorship rate was 92.8%. The majority of failures, particularly in younger patients, were attributed to aggressive osteolysis in the acetabulum, which required extensive allograft reconstruction or specialized implants for revision surgery.

The rate of dislocation as a cause for revision in patients who received a retentive cemented cup is comparable to the literature results in patients aged 70 years and older. However, in younger and more active patients, the rate of dislocation is higher compared to nonretentive cemented cups. Our institution used retentive cemented cups in all patients between 2005 and 2012, which may account for the higher rate of dislocation as a reason for revision in our patient population.

Restoring the rotation center is critical for implant longevity and motion [14], and many revision cases require the use of special augments to achieve this goal, resulting in increased risks and costs associated with revision surgery. In patients aged 70 years or older, the 10-year survival rate is comparable to unconstrained implants, and the dislocation rate is significantly lower compared to younger patients. Studies have demonstrated that using larger head implants can reduce revision rates for dislocation. Zijlstra et al. reported that for all approaches, using 32 mm heads instead of 22 to 28 mm heads is advantageous, and for the posterolateral approach, 36 mm heads can further decrease the risk of revision for dislocation [15]. Additionally, larger heads have been shown to reduce impingement, which is a potential source of instability for small-diameter heads, according to Waddell [16]. A combination of a 45° inclination of the cup and a diameter of the femoral head larger than 22 mm can minimize the risk of implant failure due to wear [17].

The Norwegian Arthroplasty Register found that femoral head size was a significant risk factor for dislocation as a cause of revision among 42,987 primary operations from 1987 to 2000 [18]. Plate et al. [19] found that the use of 28 mm femoral heads led to a higher rate of revision compared to 32 mm heads. Large-diameter femoral head articulations may reduce dislocation rates in high-risk patients without compromising functional improvements and safety. Therefore, the preoperative evaluation of patients undergoing primary THA should include the careful identification of all risk factors for dislocation. Patients with two or more risk factors for dislocation may benefit from the use of large-diameter heads rather than a retentive cup as a primary implant. 

Additionally, ceramic-on-ceramic (ZrO_2_-on-ZrO_2_) coupling has been shown to have the best performance in reducing the risk of primary postoperative failure, making it a more suitable option for young, active patients [20].

A comprehensive literature review on the results of dual mobility implants and constrained acetabular liners in revision THA was performed by Van Eecke [21]. He concluded that the use of dual mobility cups seems more appropriate since they offer lower rates of dislocations, loosening, and re-revisions in the short- and midterm. The constrained acetabular liners group had lower midterm survival rates (81.0% vs. 94.7%), higher dislocation rates (11.0% vs. 2.6%), and higher acetabular loosening rates (2.0% vs. 1%). The current study results revealed a survival rate of 92.2% at 10 years. When analyzed by age groups, below 70 years the survival rate dropped to 88.5%. The Van Eecke study was conducted for constrained implants in revision settings, compared to ours where the constrained implant was used as a primary implant. That is the reason why the survivorship is slightly lower in his study. 

Another drawback of this implant was the higher rate of loosening and revision and the extent of acetabular damage. We noticed that in the majority of revisions (55%) the bone loss was classified as Grade III Paprosky. This has great importance in terms of revision after a failed constrained implant. Although the failure rate does not seem very high, bone damage, in most cases, is important and will impact the complexity and outcome of the revision surgery tremendously.

Nowadays, we advocate for using dual mobility liners or tripolar-constrained implants for cases with expected instability after total hip arthroplasty or revision with expected instability. The improved arc of movement with dual mobility liners, minimizing impingement, makes them preferable. Constrained implants may be beneficial for global abductor deficiency or severe joint laxity [22]. 

When using constrained implants, impingement forces are dissipated through the locking mechanism to the cup and to cup–bone interfaces. This could lead to wear and loosening more rapidly than in other designs [23]. Dual mobility liners show good overall survival and a low rate of dislocation when used in cases of instability [24,25].

In adherence to our research protocol, it is essential to underscore that none of the cases under investigation involved the utilization of antibiotic cement. This deliberate omission of antibiotic cement application is pivotal in the assessment of the mechanical properties inherent to the construct. By refraining from introducing antibiotic elements into the cement, we aimed to isolate and specifically evaluate the inherent mechanical characteristics of the construct under scrutiny. This methodological decision ensured a focused examination of the primary structural attributes, facilitating a more nuanced understanding of the construct’s performance without the confounding effects of antibiotic influences [26]. Consequently, any observed alterations or improvements in mechanical properties can be attributed solely to the intrinsic nature of the construct itself, devoid of external factors such as antibiotic augmentation.

Another study reports the catastrophic failure of tripolar-constrained implants. It was demonstrated that the backside of the inserts suffered severe polyethylene deformation, wear, and scratching due to dissociation from the locking mechanism. The authors recommend conversion to a modular dual mobility liner [27]. Also, a cemented dual mobility cup is a valuable option as a primary implant for elderly patients with high-risk instability [28]. Our study retrospectively analyzed the use of semi-retentive cups as a primary method for all cases, regardless of dislocation risks. This approach is no longer supported by current evidence-based practices and should not be recommended. At the time, however, limited information was available regarding the drawbacks associated with this method. It is important to note that medical protocols and knowledge evolve over time, and what was considered standard practice at one point may not be considered best practice in the present. As medical professionals, we must stay up-to-date with the current research and guidelines to provide the best possible care for our patients [29].

This study is subject to certain limitations. Despite the substantial overall sample size, there is a notable decrease in numbers, particularly within age groups under 49 years. The inclusion criteria primarily focused on economically motivated patient selection for the studied implant, resulting in suboptimal survivorship outcomes. The contemporary landscape suggests diminished likelihood of employing this implant for primary arthroplasty, potentially diminishing the current relevance of the study’s objective. Nevertheless, the findings may hold significance in specific cases or when considering the use of analogous implants. 

## 5. Conclusions

The semi-constrained cup, when used as a primary acetabular implant, demonstrates lower survivorship rates compared to other primary implants, particularly in younger patients, due to the risk of aseptic loosening. A major contributor to this outcome is osteolysis, which results in the significant loss of acetabular bone. Therefore, the use of the semi-constrained cup as a primary implant in young and active patients without any known dislocation risk factors should be avoided due to the increased risk of loosening and acetabular osteolysis. Conversely, this design could be a viable option for elderly patients with low-activity lifestyles, muscular insufficiency, and reduced compliance with post-hip arthroplasty restrictions, as it could reduce dislocation rates without significantly impacting survivorship rates. In addition, it is important to carefully evaluate patient characteristics and risk factors before selecting an appropriate implant for primary total hip arthroplasty. One should take into consideration factors such as age, activity level, pre-existing medical conditions, and anatomical considerations when selecting an implant. Furthermore, long-term follow-up and postoperative monitoring are crucial for detecting any potential complications or adverse events associated with the selected implant. Long-term studies must be conducted to assess the potential late complications of the implant.

## Figures and Tables

**Table 1:** Demographic characteristics.

Characteristic	N (%)
**Age (mean ± SD)**	69.8 (10.3)
**Age Groups**	
0–39 years	8 (1.5%)
40–49 years	13 (2.5%)
50–59 years	55 (10.4%)
60–69 years	157 (29.8%)
70–79 years	218 (41.4%)
≥80 years	76 (14.4%)
**Gender**	
Women	378 (71.7)
Men	149 (28.3)

**Table 2:** Reasons for revision.

Reason for Revision	Percentage	Time to Revision
Cup Loosening	65%	2–10 years
Femoral Loosening	15%	2–10 years
Dislocations	12.8%	Less than 2 years
Pain	5.2%	2–10 years
Infection	2%	Less than 2 years

## Data Availability

Data are contained within the article.
